# Stability of Golden redfish (*Sebastes marinus*) during frozen storage as affected by raw material freshness and season of capture

**DOI:** 10.1002/fsn3.648

**Published:** 2018-04-16

**Authors:** Huong Thi Thu Dang, María Gudjonsdóttir, Magnea G. Karlsdóttir, Minh Van Nguyen, Tumi Tómasson, Sigurjon Arason

**Affiliations:** ^1^ Faculty of Food Science and Nutrition University of Iceland Reykjavik Iceland; ^2^ Faculty of Food Technology Nha Trang University Nha Trang Vietnam; ^3^ Matís ohf./Icelandic Food and Biotech R&D Reykjavik Iceland; ^4^ United Nations University Fisheries Training Programme Reykjavik Iceland

**Keywords:** frozen storage, Golden redfish, light/dark muscle, physicochemical properties, processing delay, seasonal variation

## Abstract

Physicochemical changes of Icelandic golden redfish (*Sebastes marinus*) as affected by seasonal variation (June and November) and raw material freshness (processed 4 and 9 days postcatch) during frozen storage (at −25°C for 20 months) were studied to find optimal conditions for production of high‐quality frozen products. Thawing loss, cooking yield, and color of the fillets as well as chemical composition, water holding capacity, pH, total volatile basic nitrogen, lipid oxidation, and hydrolysis of the light and dark muscle were analyzed every 4 months of frozen storage. Lipid hydrolysis was the main degradation process in the light muscle, while the dark muscle was more affected by lipid oxidation. Fish caught in November showed greater instability in the analyzed parameters during storage than fish caught in June, which could be linked to differences in individual poly unsaturated fatty acids between the two seasons. The quality attributes of fish processed on day 9 were similar to fish processed 4 days postcatch, except slightly higher thawing loss and yellowness, were observed in fish processed 9 days postcatch. Stability of golden redfish through frozen storage was higher in the fish caught in June than in November.

## 1 INTRODUCTION

Frozen storage is an efficient preservation method to maintain the quality and extend the shelf life of fishery products. However, some deterioration in fish quality still occurs during frozen storage, which results in quality loss and limits the storage life of the product. Quality deterioration has been mainly associated with lipid hydrolysis and oxidation, as well as protein denaturation, which all affects sensory quality, nutritional value, texture changes, yield of thawing and cooking, and ability of the fish muscle to retain its natural water (Erickson, 1997). Quality and storage life of frozen fish can be influenced by several factors, such as the chemical composition of the raw material (Romotowska, Karlsdóttir, Gudjonsdóttir, Kristinsson, & Arason, 2016), temperature and storage time of raw material before freezing (Giannini, Parin, Gadaleta, Carrizo, & Zugarramurdi, 2001; Lauder, MacCallum, & Idler, 1970), as well as conditions during frozen storage and transportation (Dang et al., 2017).

The chemical composition of several fish species undergoes seasonal variation (Hamre, Liea, & Sandnes, 2003; Huss, 1995), which influences quality degradation. Rancidity in cod fillets is greater in winter and early spring than during the summer and fall (Castell & MacLean, 1964). Seasonal variation has been shown to affect the total lipids content and lipid hydrolysis of frozen hake fillets (Roldán, Roura, Montecchia, Borla, & Crupkin, 2005), and rancidity development of frozen mackerel (Romotowska et al., 2016). According to Aubourg, Lago, Sayar, and González (2007), higher peroxide formation was observed during frozen storage of blue whiting and hake captured in May than in November. Giannini et al. (2001) stated that under the same storage conditions (8 days on ice postcatch), total volatile basic nitrogen (TVB‐N) from hake captured in summer was 66% higher than for hake captured in winter. Moreover, the chemical composition varies widely between the different parts of the fish and muscle types, leading to a variation in the quality degradation rate within the fillet, for example does rancidity develop faster in the dark muscle compared to the light muscle in several fish species (Dang et al., 2017; Karlsdóttir et al., 2014; Undeland, Ekstrand, & Lingnert, 1998).

Raw material treatment and storage prior to further processing can furthermore affect the quality and stability of a product. Lauder et al. (1970) reported that redfish, which had been iced for 2 days prior to being processed, frozen, and stored at −18°C, were of acceptable quality for 83–94 weeks, while those kept on ice for 12 days prior to freezing had a storage life of only 51 weeks.

Golden redfish (*Sebastes marinus*) is one of the most common and commercially important fish species in Icelandic waters. The catch of golden redfish increased from 39,000 tonnes in 2011 to 57,900 tonnes in 2015 (Statistics Iceland, 2016), and the economic importance of the fishing has thus increased. Previously, redfish was mainly processed into fresh fillets for export. However, production of frozen redfish fillets has increased to adapt to the increased catching capacity (Iceland Responsible Fisheries, 2016). One of the main challenges lies in retaining quality of the raw material due to the long duration of fishing trips and thus also processing delays. Frozen redfish fillets are commonly processed up to 9 days after catch in the Icelandic fishing industry. While awaiting processing and freezing, the raw materials are iced in tubs or coolers to maintain the temperature of the fish at 0°C. In order to understand how processing delays affect the quality and stability of the final product during frozen storage, analysis of physicochemical properties changes through storage are essential.

Moreover, golden redfish is caught in the Icelandic fishing zone all year round, but the largest catches are often obtained in late winter and early spring (Ministry of Industries and Innovation, 2017). The mating season of golden redfish is from September to November, while spawning occurs between April and June, but is at a maximum in May (Matis, 2015). Therefore, analyses on chemical composition and quality changes of golden redfish during frozen storage when fish are caught in the mating (November) and after spawning (June) seasons would benefit the Icelandic golden redfish industry in order to optimize their capture and production. To the best of our knowledge, few studies have been conducted on these issues.

This study therefore aimed at investigating the effect of seasonal variation and raw material freshness before freezing on the physicochemical properties of the light and dark muscle of golden redfish (*S. marinus*) fillets during frozen storage.

## 2 MATERIALS AND METHODS

### 2.1 Raw materials, processing, and sampling

Icelandic golden redfish (*S. marinus*) were caught off the southwest coast of Iceland in June and November 2015 by fresh fish trawlers. After catching, the fish were immediately put in 460‐L plastic tubs (Promens, Dalvik, Iceland) with ice to maintain the temperature of the fish on‐board at 0°C (100 kg of ice/300 kg fish). After landing, 4 days postcatch, the fish were transported to the factory for processing and divided into two treatments, one processed 4 days postcatch, while the other was processed 9 days postcatch. All samples were processed as skinless fillets, frozen in layers in 5 kg/pack (4 layers, separated by polyethylene sheets) in a plate freezer (Jackstone, UK), and were then packed in waxed cardboard boxes, and finally stored at −25°C.

Analyses were performed after 0, 4, 8, 12, 16, and 20 months of frozen storage at −25°C. Prior to analysis, one 5‐kg pack from each treatment was thawed at a refrigerated temperature of 4 ± 1°C for at least 24 hr. After thawing, fish from each treatment were divided into three groups (*n *=* *3), each containing ten fillets (average weight per fillet was 120 ± 20 g). Light and dark muscles of the fillets were manually separated, then minced, and used for all analyses. Chemical analyses were performed separately on the light and the dark muscle for each group. Water, total lipids (TL), phospholipids (PL), fatty acid composition, lipid oxidation and hydrolysis products, and pH were performed on both muscle types, while protein content, water holding capacity (WHC), and total volatile basic nitrogen (TVB‐N) analyses were performed only on the light muscle. Analyses were set up in triplicate for muscle pH, PL content, peroxide values (PV), and thiobarbituric acid reactive substances (TBARS), while analyses for WHC, TVB‐N, protein, water, TL content, free fatty acids (FFA), and fatty acid composition were performed in duplicates. Thawing loss, color, and cooking yield (CY) analyses were performed on individual fillets (5 fillets for each group). The weight proportion of muscle types (light and dark) of the fillets was performed at month 0 of storage for each fillet (*n *=* *15).

All chemicals used in this study were purchased from Sigma‐Aldrich (Steinheim, Germany) and Fluka (Buchs, Switzerland).

### 2.2 Thawing loss, WHC, and CY

Thawing loss of the fillets was calculated as the ratio (%) of liquid lost during thawing to the weight of the frozen fillets as presented by Equation (1):

(1) $$\mathrm{Thawing}\;\mathrm{loss}\left(\%\right)=\frac{\mathrm{weight}\;\mathrm{of}\;\mathrm{frozen}-\mathrm{weight}\;\mathrm{of}\;\mathrm{thawed}}{\mathrm{weight}\;\mathrm{of}\;\mathrm{frozen}}\times 100$$

Water holding capacity of the light muscle was determined by a centrifugation method described by Eide, Borresen, and Strom (1982). Approximately 2 g of the minced sample was weighed into a vial and centrifuged at 1,350 rpm for 5 min at 4°C. The WHC (%) was calculated as the ratio of the remaining liquid compared to the liquid content in the sample before centrifugation.

Cooking yield of the fillets was calculated as the ratio (%) of the sample weight after cooking to the weight of the sample prior to cooking as presented by Equation (2). About 35 g of each fillet was weighed and heated in a steaming oven (Convotherm OGS 6.10 Combi convection steam oven; Elektrogeräte GmbH, Eglfing, Germany) at 100°C for 10 min. Samples were drained for 10 min prior to being reweighed.

(2) $$\mathrm{CY}\left(\%\right)=\frac{\mathrm{weight}\;\mathrm{of}\;\mathrm{cooked}\;\mathrm{sample}}{\mathrm{weight}\;\mathrm{of}\;\mathrm{sample}\;\mathrm{before}\;\mathrm{cooking}}\times 100$$

### 2.3 Color

The color of the samples was determined with a Minolta Chroma Meter CR‐400 (Minolta, Osaka, Japan) using the CIE Lab system. The instrument recorded the *L‐*value, indicating lightness on the scale from 0 to 100 from black to white, the *a*‐value, ranging from (+) red to (−) green, and the *b‐*value, ranging from (+) yellow to (−) blue. The color was measured above the lateral line at five positions, from the head to the tail of each fillet on the light muscle side of the fillet.

### 2.4 Weight proportion, proximate composition, and PL content

The weight proportions were calculated as the ratio (%) of the dark and light muscle weight compared to the weight of the whole fillet. The water content of the samples was determined as the weight loss during drying at 102–104°C for 4 hr according to ISO‐6496 (1999). Results were calculated as a percentage of the wet muscle. TL was extracted according to the Bligh and Dyer (1959) method and results expressed as a percentage of the wet muscle. Protein content was determined according to ISO 5983‐2 (2009) and expressed as a percentage of the wet muscle. PL content was determined on the TL extracts and was measured using a colorimetric method as described by Stewart (1980), based on the formation of a complex between phospholipids and ammonium ferrothiocyanate, followed by reading the absorbance of the resultant solution at 488 nm (UV‐1800 spectrophotometer; Shimadzu, Kyoto, Japan). Phosphatidylcholine in chloroform (1 mg/ml) was prepared for a standard curve, and results were expressed as g PL per 100 g TL.

### 2.5 Fatty acid profile

The fatty acid composition was determined on the TL extracts by gas chromatography (Varian 3900 GC; Varian, Inc., Walnut Creek, CA, USA) of fatty acid methyl esters, based on the AOAC 996.06 (2001) method. The fatty acid methyl esters were separated on a Varian 3900 GC equipped with a fused silica capillary column (HP‐88, 100 m × 0.25 mm × 0.20 μm film; Agilent Technologies), split injector, and flame ionization detector fitted with a Galaxie Chromatography Data System (Version 1.9.3.2 software). The setting of the oven was as follows: 100°C for 4 min, then increased to 240°C at a rate of 3°C/min, and held at this temperature for 15 min. The injector and detector temperatures were 225 and 285°C, respectively. Helium was used as the carrier gas, and the column flow rate was set to 0.8 ml/min with a split ratio of 200:1. The methylation of fatty acids was carried out according to the AOAC Ce 1b‐89 method (1998), and results expressed as g fatty acids per 100 g TL.

### 2.6 Muscle pH and TVB‐N

The muscle pH was measured by inserting the pH probe (Radiometer PHM80 Portable pH meter, Denmark) directly into the muscle samples.

Total volatile basic nitrogen was determined in the light muscle based on the method of Malle and Poumeyrol (1989). The boric acid solution turns green when alkalinized by the distilled TVB‐N, which was titrated with aqueous 0.025 N sulfuric acid solution using a 0.05 ml graduated buret. Results were expressed as mg N/100 g muscle.

### 2.7 Lipid oxidation

Peroxide values expressed as μmol lipid hydroperoxide per kg of wet muscle (μmol/kg muscle), were determined based on the method of Shantha and Decker (1994), with modifications. Each sample (5.0 g) was homogenized (Ultra‐Turrax T‐25 basic, IKA, Germany) for 20 s with 10 ml of an ice‐cold chloroform:methanol (1:1) solution, containing 500 ppm butylated hydroxytoluene (BHT). Sodium chloride (5.0 ml; 0.5 mol/L) was then added to the mixture and homogenized for 20 s. Phase separation was carried out by centrifugation at 5,525 g for 5 min at 4°C (TJ‐25 Centrifuge, Rotor TS‐5.1‐500; Beckmann Coulter, USA). The lower chloroform layer containing lipids was collected (0.5 ml for the dark, and 0.8 ml for the light muscle) and matched with 0.5 and 0.2 ml of the chloroform:methanol (1:1) solution for the dark and light muscle, respectively. Finally, a total amount of 5‐μl mixture (1:1) of ammonium thiocyanate (4 mol/L) and ferrous chloride (80 mmol/L) was added before vortexing. The absorbance was then measured at 500 nm (Tecan Sunrise, Switzerland) on a polypropylene microplate (Eppendorf, microplate 96/F‐PP) after 10 min of incubation at room temperature. A standard curve was prepared from cumene hydroperoxide.

Thiobarbituric acid reactive substances expressed as μmol malondialdehyde diethyl acetal per kg of wet muscle (μmol MDA/kg muscle) were determined based on Lemon (1975) with modifications. Each sample (5.0 g) was homogenized with 10.0 ml of trichloroacetic acid (TCA) extraction solution (7.5% TCA, 0.1% propyl gallate, and 0.1% ethylenediaminetetraacetic acid [EDTA] in a phosphate buffer mixture prepared in ultrapure water) using a homogenizer (Ultra‐Turrax T‐25 basic; IKA) at 500 rpm for 20 s. After centrifugation at 5,100 rpm for 20 min at 4°C (TJ‐25 Centrifuge, Rotor TS‐5.1‐500; Beckmann Coulter), the collected supernatant was filtered with a Whatman qualitative filter paper no. 4. Thiobarbituric acid (0.02 mol/L) in an amount of 0.5 and 0.2 ml were mixed with the collected supernatant, 0.5 ml for dark and 0.8 ml for light muscle, respectively, before heating the mixture in a water bath at 95°C for 40 min. After heating, the mixture was immediately placed on ice for cooling, and then, 200 μl of the sample were loaded into 96‐well microplates (NUNC A/S Thermo Fisher Scientific, Roskilde, Denmark). The absorbance was measured at 530 nm (Tecan Sunrise). A standard curve was prepared from 1.1.3.3‐tetraethoxypropane.

### 2.8 Lipid hydrolysis

Free fatty acids content was determined on the TL extracts by the Lowry and Tinsley (1976) with a modification made by Bernardez, Pastoriza, Sampedro, Herrera, and Cabo (2005). The absorbance of the solution was read at 710 nm (UV‐1800 spectrophotometer). A standard curve was prepared from oleic acid in a concentration range of 2–14 μmol. Results were expressed as g FFA per 100 g TL.

### 2.9 Data analysis

Statistical analyses were carried out using the STATISTICA software (Version 10.0; StatSoft, OK, USA) and Microsoft Office Excel 2013 (Microsoft Inc., Redmond, WA, USA). One‐way ANOVA, Tukey HSD's test, and Student *t* tests for independent samples were performed on the means (*n *=* *3) for each treatment. Pearson's correlation analysis was carried out to find the correlations between variables. The significance of difference was defined as *p *<* *.05 for all analyses. A Principal Components Analysis (PCA) was carried out using Unscrambler (Version 10.2; CAMO ASA, Trondheim, Norway) to identify similarities and differences between samples. All variables were weighed with the inverse of the standard deviation to correct for different scales of the variables.

## 3 RESULTS AND DISCUSSION

### 3.1 Thawing loss, WHC, and CY

**Figure 1:**
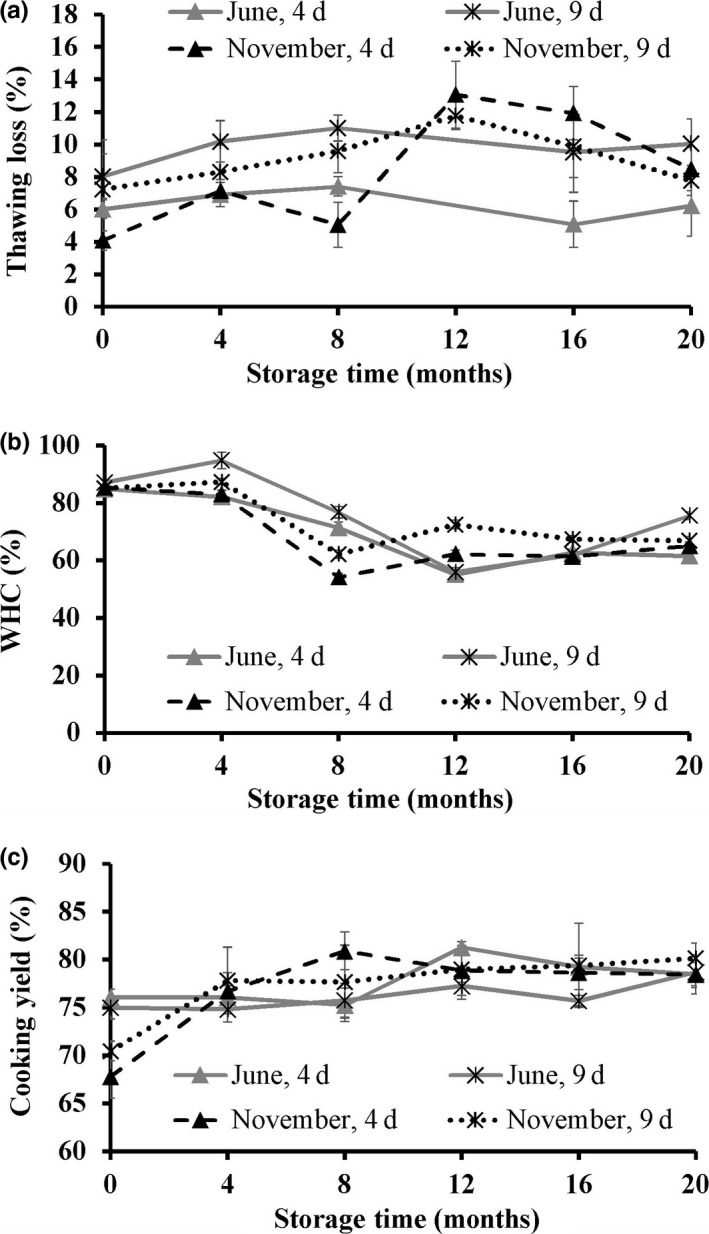
Thawing loss (%) (a), water holding capacity (WHC; %) (b), and cooking yield (%) (c) of golden redfish fillets as affected by the seasonal variation (June and November), and raw material freshness (processed 4 and 9 days postcatch) through frozen storage at −25°C (*n *=* *3, mean ± standard deviation)

Thawing loss of fillets from fish caught in June was fairly stable throughout the storage period, while an increase in thawing loss was observed for the fish caught in November (Figure 1a). The increase in thawing loss of the redfish fillets was considered to be mainly due to protein denaturation (Xiong, 1997) and cell rupturing caused by ice crystal formation. Processing of raw material of lower freshness (on day 9) led to a slightly higher thawing loss compared to fillets processed and frozen 4 days postcatch for both seasons. The higher thawing loss observed in the fish processed on day 9 can also be assumed to be mainly due to higher protein denaturation that occurs during iced storage, as freshness affects the biochemical properties of fish muscle (Kim & Park, 2007).

The ability of muscles to retain water is an essential quality parameter, and a high WHC is of great importance both to the industry and the consumers. The WHC of the light muscle was stable during the first 4 months, followed by a decrease until month 8 and month 12 of storage for fish caught in November and June, respectively (Figure 1b). The WHC then remained stable to the end of the study for all treatments. The decrease in WHC on the early stages of storage can likely be explained by the protein denaturation in the fish muscle occurring during frozen storage, which in turn led to loss of functional properties of the proteins and caused a decrease in WHC. Moreover, enzymatic activities (lipase, phospholipase, and protease) in the light muscle are also thought to contribute to the decrease in WHC during frozen storage (Xiong, 1997).

The CY is related to the liquid lost during cooking and comes from constitutive water as a result of protein denaturation and from the fat which melts during heating. In the present study, CY increased during the first 8 months of storage for all treatments (Figure 1c). A significant negative correlation (*r *=* *−.64) was obtained between the WHC and the CY of the fillets. No significant differences were observed in the CY neither due to the raw material freshness nor the season of capture.

### 3.2 Color

**Figure 2:**
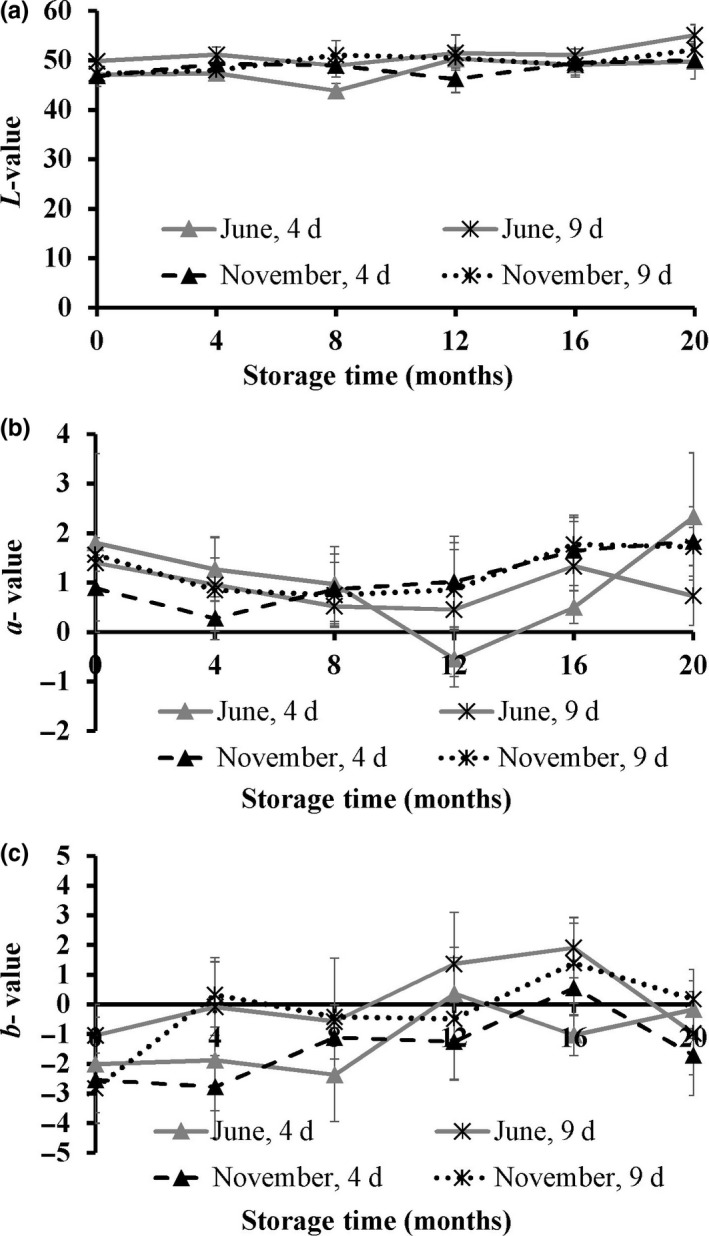
Color *L*‐value (a), *a*‐value (b), and *b*‐value (c) of golden redfish fillets as affected by the seasonal variation (June and November), and raw material freshness (processed 4 and 9 days postcatch) through frozen storage at −25°C (*n *=* *3, mean ± standard deviation)

Lightness values of the golden redfish fillets were stable during the frozen storage for all treatments. Furthermore, no significant differences were observed in the *L*‐values neither due to freshness nor catching season (Figure 2a). The *a*‐value of the fillets processed 4 days postcatch in June fluctuated slightly, but in general, the *a*‐value of the fillets was stable for both seasons (Figure 2b). In general, the *b*‐value increased during frozen storage, indicating a more yellow appearance of the fillets with storage. Furthermore, slightly higher *b*‐values were observed in the fish processed 9 days postcatch than in the fish processed 4 days postcatch (Figure 2c), in agreement with the lower freshness of the fish processed 9 days postcatch.

### 3.3 Weight proportions, proximate composition, and PL content

On average, the light muscle constituted 88.1 ± 2.6% of the fish fillets, independent of the season of catch. The water content in the light and dark muscle of the fish caught in June (analyzed 4 days postcatch) was 80.2 ± 0.3% and 75.4 ± 0.3%, respectively. However, significantly higher water content was observed in the fish caught in November, as represented by a water content of 81.4 ± 0.2% and 77.7 ± 0.4%, in the light and dark muscle, respectively. The TL content of the light muscle was significantly lower than in the dark muscle of fish caught in both seasons. However, no significant differences were observed in the TL content due to the season of catch. The protein content of the light muscle was 18.2 ± 0.1% in June and 18.9 ± 0.1% in November. According to Gislason and Astthorsson (1995), the spring bloom of the phytoplankton and total zooplankton in the southwest of Iceland are low in the winter and reach a maximum during the summer (May–June). In this study, golden redfish were caught off the southwest coast of Iceland during the first weeks of June and November. These could explain that even though the golden redfish were caught in the late spawning season (June), the protein and TL content of the fish muscle did not differ to fish in the mating season (November) due to heavy feeding.

**Figure 3:**
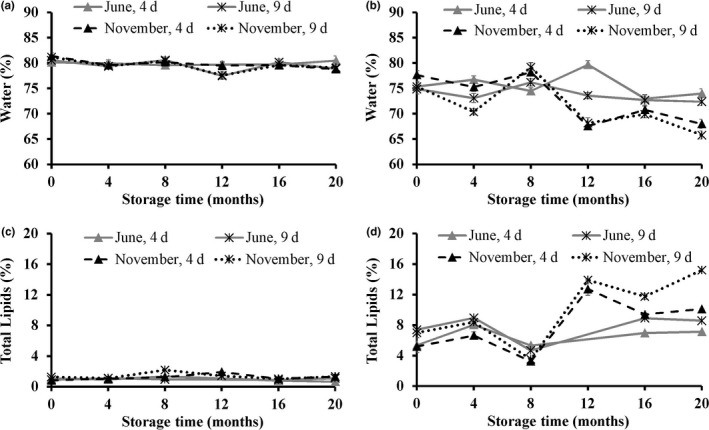
Water content (percentage of the wet muscle) in the light (a) and the dark (b) muscle, and total lipids content (percentage of the wet muscle) in the light (c) and the dark (d) muscle of golden redfish, as affected by the seasonal variation (June and November), and raw material freshness (processed 4 and 9 days postcatch) through frozen storage at −25°C (*n *=* *3, mean ± standard deviation)

The water content in the light muscle was both higher, and more stable during frozen storage compared to the dark muscle of both seasons and the raw material freshness upon processing and freezing (4 or 9 days postcatch) (Figure 3a,b). Similar results have been obtained for frozen saithe and hoki (Karlsdóttir et al., 2014), who showed that no significant changes in water content during frozen storage. The water content in the dark muscle of the golden redfish caught in June was initially stable but then tended to decrease after 12 months of frozen storage. However, for redfish caught in November, the water content of the dark muscle fluctuated throughout storage and exhibited a lower water content than the fish caught in June. The water and TL content were inversely correlated (*r *=* *−.96) throughout storage and constituted together around 80.0%–82.0% of the wet muscle, which is in agreement with the findings of Karlsdóttir et al. (2014) on the composition of saithe and hoki. The TL content of the light muscle was stable, but fluctuated heavily in the dark muscle during frozen storage. Higher TL values were generally observed in the golden redfish caught in November compared to fish caught in June (Figure 3c,d). However, the protein content was stable during the frozen storage of both raw material freshness states upon processing and season of catch.

**Figure 4:**
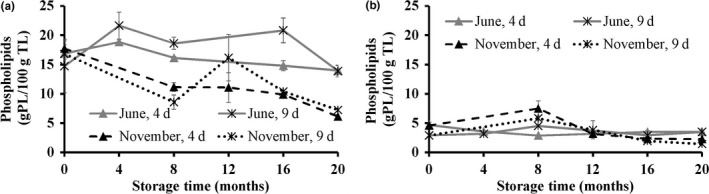
Phospholipids content (g PL/100 g TL) in the light (a) and the dark (b) muscle of golden redfish, as affected by the seasonal variation (June and November), and raw material freshness (processed 4 and 9 days postcatch) through frozen storage at −25°C (*n *=* *3, mean ± standard deviation)

The PL content was significantly higher in the light muscle than in the dark muscle (Figure 4) in agreement with previous studies of saithe and hoki (Karlsdóttir et al., 2014), and herring (Dang et al., 2017). No significant differences were observed in the PL content of the light muscle due to raw material freshness. However, a higher PL content was generally observed in the light muscle of fish caught in June than in November. The PL content in the light muscle of fish caught in June increased during the first 4 months of storage, which may be attributed to increased extractability of PL, resulting from protein denaturation during storage (Kolakowska, Olley, & Dunstan, 2003). However, after 4 months of storage, the PL content decreased for the remaining duration of storage. On the other hand, the PL content of the light muscle of fish caught in November decreased throughout the frozen storage. This decrease in PL content in both seasons after long‐term frozen storage was expected due to the hydrolytic activities by phospholipases (Sista, Erickson, & Shewfelt, 1997). No significant changes were observed in PL content in the dark muscle, independent of season of catch.

### 3.4 Fatty acid profile

Polyunsaturated fatty acids (PUFA) were dominant in the fatty acid profile in the golden redfish light muscle of the raw material of both seasons, followed by monounsaturated fatty acids (MUFA), and finally saturated fatty acids (SFA) (Table 1). However, in the dark muscle, the amounts of MUFA were the highest, followed by PUFA, and lastly SFA. These findings were similar to the previous observations of the fatty acid composition of hoki by Karlsdóttir et al. (2014).

**Table 1:** Fatty acid profile (g fatty acids/100 g TL) of golden redfish light and dark muscle (analyzed 4 days postcatch) caught in Icelandic waters in June and November, 2015 (*n *=* *3, mean ± standard deviation)

Fatty acids	Light muscle		Dark muscle	
	June	November	June	November
C14:0	3.4 ± 0.4^ax^	2.6 ± 0.4^bx^	5.0 ± 0.2^ay^	4.3 ± 0.3^by^
C15:0	0.3 ± 0.0^a^	0.2 ± 0.1^ax^	ND	0.3 ± 0.0^x^
C16:0	15.6 ± 0.8^ax^	15.8 ± 0.3^ax^	*12.4 ± 0.1* ^ay^	12.6 ± 0.2^ay^
C17:0	0.2 ± 0.0^ax^	0.1 ± 0.1^ax^	0.2 ± 0.0^ax^	0.1 ± 0.0^ax^
C18:0	3.0 ± 0.1^ax^	3.2 ± 0.0^ax^	2.6 ± 0.0^ay^	2.7 ± 0.1^ay^
ΣSFA	22.5 ± 1.0^ax^	21.9 ± 0.1^ax^	20.4 ± 0.9^ay^	20.1 ± 0.0^ay^
C16:1n9	3.7 ± 0.4^ax^	3.9 ± 0.1^ax^	4.9 ± 0.7^ay^	5.9 ± 0.4^by^
C18:1n5	1.3 ± 0.6^x^	*ND*	2.4 ± 0.8^y^	*ND*
C18:1n7	4.2 ± 1.2^ax^	2.7 ± 0.0^bx^	5.7 ± 3.6^ay^	3.3 ± 0.2^by^
C18:1n9	7.0 ± 1.3^ax^	9.7 ± 0.2^bx^	8.1 ± 4.6^ay^	13.1 ± 1.4^by^
C20:1n7	4.7 ± 0.5^ax^	7.8 ± 0.2^bx^	9.0 ± 2.1^ay^	11.6 ± 0.2^by^
C20:1n9	0.9 ± 0.7^ax^	0.3 ± 0.1^ax^	2.9 ± 1.8^ay^	0.4 ± 0.2^bx^
C20:1n11	1.4 ± 0.7	*ND*	*ND*	0.8 ± 0.1
C22:1n9	7.9 ± 2.2^ax^	7.7 ± 0.2^bx^	13.0 ± 1.1^ay^	11.9 ± 0.7^by^
C22:1n11	2.1 ± 0.9^ax^	1.1 ± 0.0^bx^	2.6 ± 1.1^ay^	1.7 ± 0.2^by^
C24:1	0.6 ± 0.1^ax^	0.2 ± 0.0^bx^	2.0 ± 1.2^ay^	0.3 ± 0.0^bx^
ΣMUFA	32.5 ± 3.2^ax^	33.5 ± 0.2^ax^	49.5 ± 0.0^ay^	48.4 ± 0.6^by^
C16:3n3	0.6 ± 0.0^x^	*ND*	0.5 ± 0.3^x^	*ND*
C18:3n3	1.3 ± 0.1^ax^	0.4 ± 0.0^bx^	1.7 ± 0.6^ay^	0.5 ± 0.0^by^
C18:4n3	1.0 ± 0.1^ax^	0.7 ± 0.3^ax^	1.3 ± 0.0^ay^	1.2 ± 0.4^by^
C20:4 n3	*ND*	0.4 ± 0.0^x^	*ND*	0.5 ± 0.0^x^
C22:5 n3	*ND*	0.4 ± 0.0^x^	*ND*	0.3 ± 0.3^x^
C20:5n3 (EPA)	7.9 ± 0.3^ax^	8.3 ± 0.2^bx^	6.5 ± 1.6^ay^	7.3 ± 0.0y^a^
C22:6n3 (DHA)	23.5 ± 1.1^ax^	26.3 ± 0.6^bx^	11.5 ± 1.3^ay^	14.5 ± 0.0^by^
Σn−3	34.2 ± 0.6^ax^	36.5 ± 0.7^bx^	21.4 ± 0.0^ay^	24.3 ± 0.4^by^
C18:2n6	1.7 ± 0.2^ax^	1.6 ± 0.1^ax^	1.5 ± 0.2^ax^	1.5 ± 0.0^bx^
C20:4n−6	*ND*	1.2 ± 0.0^x^	*ND*	0.7 ± 0.1^y^
C22:5n6	1.1 ± 0.2^ax^	0.9 ± 0.0^ax^	1.4 ± 0.9^ax^	0.8 ± 0.1^bx^
Σn−6	2.7 ± 0.4^ax^	3.7 ± 0.1^bx^	2.9 ± 1.1^ax^	3.1 ± 0.1^by^
C16:2n4	0.4 ± 0.1^ax^	0.2 ± 0.0^bx^	0.8 ± 0.0^ay^	0.2 ± 0.0^bx^
C20:4	0.5 ± 0.1^x^	*ND*	0.6 ± 0.0^x^	*ND*
ΣPUFA	37.9 ± 0.1^ax^	40.4 ± 0.7^bx^	25.7 ± 1.0^ay^	27.6 ± 0.5^by^
n−3/n−6	12.7 ± 2.2^ax^	9.8 ± 0.3^bx^	7.9 ± 2.9^ay^	8.0 ± 0.2^ay^

a, b: different superscript letters in each row indicate a significant difference within a variable between seasons (June vs. November) in the same muscle type (light or dark muscle).

x, y: different superscript letters in each row indicate a significant difference within a variable between muscle types (light vs. dark) within the same season of catch.

ND: not detected.

The major SFA in both muscle types and seasons was palmitic acid (C16:0), while erucic acid (C22:1*n*−9) and oleic acid (C18:1*n*−9) were the predominant fatty acids amongst the MUFAs. Significantly higher amounts of oleic acid were observed in both muscle types of fish captured in November compared to fish caught in June. Amongst the PUFAs, docosahexaenoic acid (DHA, C22:6*n*−3) predominated the profile, followed by eicosapentaenoic acid (EPA, C20:5*n*−3). EPA and DHA levels were higher in the light muscle than in the dark muscle of the redfish at both seasons. Furthermore, a higher *n*−3/*n*−6 ratio was obtained in the light muscle compared to the dark muscle of fish caught during both seasons. These results indicated that the light muscle can be considered more valuable than the dark muscle, as the *n*−3/*n*−6 ratio is a good index for comparing the relative nutritional value of fish oils (Pigott & Tucker, 1990), but a higher *n*−3/*n*−6 fatty acid ratio in the human dietary has been shown to have positive traits toward preventing coronary heart disease and in reducing cancer risk (Kinsella, Lokesh, & Stone, 1990).

Generally, no significant differences were observed due to season of catch in the amount of SFAs and MUFAs of neither muscle types. However, the amount of PUFAs in the fish caught in November was significantly higher than in the fish caught in June, and especially in the amounts of EPA and DHA. These differences are thought to be mainly influenced by the season, food availability, age, and size of the fish, as well as their maturation status (Aidos, van der Padt, Luten, & Boom, 2002). The higher amount of PUFA in the fish caught in November might contribute to the higher instability of the lipid properties during frozen storage of the fish caught in November, compared to the fish caught in June. These findings were in line with the observations of Dewitt (1963), who reported that the unsaturation of cod liver oil increased steadily from summer to autumn, and reached a maximum in winter.

### 3.5 Muscle pH and total volatile basic nitrogen

The initial muscle pH was 6.6 ± 0.2 for both the light and dark muscle of fish caught in June, whereas a pH of 6.7 ± 0.1 in both muscle types in the fish caught in November. Thus, no significant difference was observed in muscle pH neither due to muscle types, raw material freshness (processed 4 and 9 days postcatch), nor season. Furthermore, the frozen storage did not result in any significant pH changes, ranging from 6.5 to 6.7 for both muscle types of fish caught in June, and from 6.6 to 6.8 for fish caught in November.

Total volatile basic nitrogen is one of the indicators of muscle freshness. The TVB‐N fluctuated during frozen storage and ranged between 11.0 and 14.0 mg N/100 g in the light muscle, independent of season and raw material freshness. These values are within the limit of acceptance, as the critical limits of TVB‐N range from 25.0 to 35.0 mg N/100 g muscle (Venugopal, 2006). These results indicate that the light muscle of the golden redfish fillets stored at −25°C was stable, and of acceptable freshness in terms of TVB‐N, during the 20 months of storage.

Frozen storage inhibited microbial activity, which corresponds basically to both pH and TVB‐N values. This could explain the low values of both pH and TVB‐N obtained in the present study.

### 3.6 Lipid oxidation and hydrolysis

Lipid oxidation was assessed by the analysis of primary (PV) and secondary (TBARS) lipid oxidation products. Significant increases in both PV and TBARS content of both muscle types were observed in all treatments during frozen storage. However, the formation rate of oxidation products in the fish fillets depended strongly on the muscle types. The dark muscle was more progressive toward lipid oxidation compared to the light muscle. This was expected due to the higher lipid content in the dark muscle compared to the light muscle (Hultin, 1994). A similar trend has been observed earlier for both lean fish (Karlsdóttir et al., 2014) and fatty fish species (Dang et al., 2017; Undeland et al., 1998).

**Figure 5:**
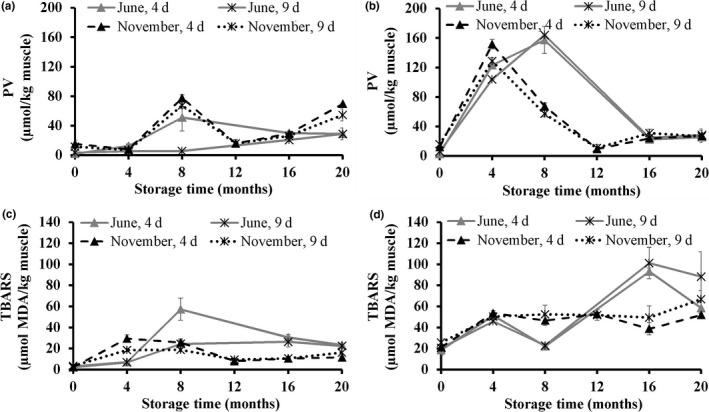
Peroxide value (PV; μmol/kg muscle) in the light (a) and the dark (b) muscle, and thiobarbituric acid reactive substances (TBARS; μmol MDA/kg muscle) in the light (c) and the dark (d) muscle of golden redfish, as affected by the seasonal variation (June and November), and raw material freshness (processed 4 and 9 days postcatch) through frozen storage at −25°C (*n *=* *3, mean ± standard deviation)

In the light muscle, the formation of PV increased only after 4 months, reaching a small peak after 8 months of frozen storage (Figure 5a). In the dark muscle, the PV content increased sharply already from the beginning of storage and reached a peak at 8 and 4 months for the fish caught in June and in November, respectively (Figure 5b). The earlier oxidation production in the dark muscle of fish caught in November is thought due to its higher PUFA content compared to fish caught in June.

The peaks in PV were followed by the decomposition of hydroperoxides to secondary oxidation products until month 16 and 12 of frozen storage for the June and November samples, respectively. This was coupled to an increase in TBARS content observed at the same storage points. The TBARS content of the light muscle increased slowly during the storage time (Figure 5c). However, the TBARS content in the dark muscle of fish caught in June fluctuated, but with an overall increasing trend during storage for fish caught at both seasons (Figure 5d). Pearson's correlation showed a significant positive correlation (*r *=* *0.53) between TBARS content and yellowness. The increase in *b*‐value is thus believed to be related to oxidation of the muscle lipids during frozen storage (Erickson, 1997), as indicated by an increasing yellow/brown nuance. A significant negative correlation (*r *=* *−.71) was obtained between the PV content and WHC of the light muscle. This indicates that lipid oxidation may influence WHC of the muscle, either directly or indirectly, through oxidative alterations of the muscle proteins.

**Figure 6:**
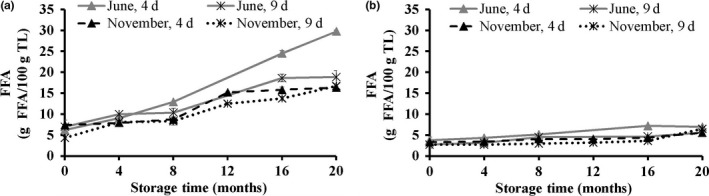
Free fatty acids content (FFA; g FFA/100 g TL) in the light (a) and the dark (b) muscle of golden redfish, as affected by the seasonal variation (June and November), and raw material freshness (processed 4 and 9 days postcatch) through frozen storage at −25°C (*n *=* *3, mean ± standard deviation)

Lipid hydrolysis may occur postmortem in fish leading to an increase in FFA content, due to increased lipase and phospholipase activities (Pacheco‐Aguilar, Lugo‐Sánchez, & Robles‐Burgueň, 2000). Higher hydrolytic activity as assessed by FFA content was observed in the light muscle compared to the dark muscle at both seasons (Figure 6). Accumulation of FFA was observed in the light muscle during storage, while the FFA formation was rather stable in the dark muscle. This can be explained by a higher PUFA content in the light muscle, making it more susceptible to lipid hydrolysis compared to the dark muscle (Polvi, Ackman, Lall, & Saunders, 1991). Faster formation of FFA in the light muscle during frozen storage may be a consequence of lipid degradation due to phospholipase and lipase activity (Aubourg & Medina, 1999; Pacheco‐Aguilar et al., 2000), which may, furthermore, explain the observed reduction of PL content in the light muscle during storage (Figure 4a).

A significant negative correlation (*r *= −0.58) was observed between the FFA and TL content. This is in agreement with the previous studies of Sheltawy and Olley (1966) for cod muscle. On the other hand, lipid hydrolysis may speed up lipid oxidation (Kaneniwa, Miao, Yuan, Iida, & Fukuda, 2000; Sista et al., 1997), which is consistent with the increase in PV and TBARS content during frozen storage as observed in the current study. Results from the present study also show a relationship between FFA content and WHC (*r *=* *−.60) in the light muscle. It can be assumed that the FFA accumulation in the light muscle affected texture deterioration of the fillet by interacting with the muscle proteins (Mackie, 1993), which led to a decrease in WHC of the light muscle during frozen storage. The formation of FFA was, however, neither affected by the season, nor the freshness of raw material.

### 3.7 Multivariate data analysis

**Figure 7:**
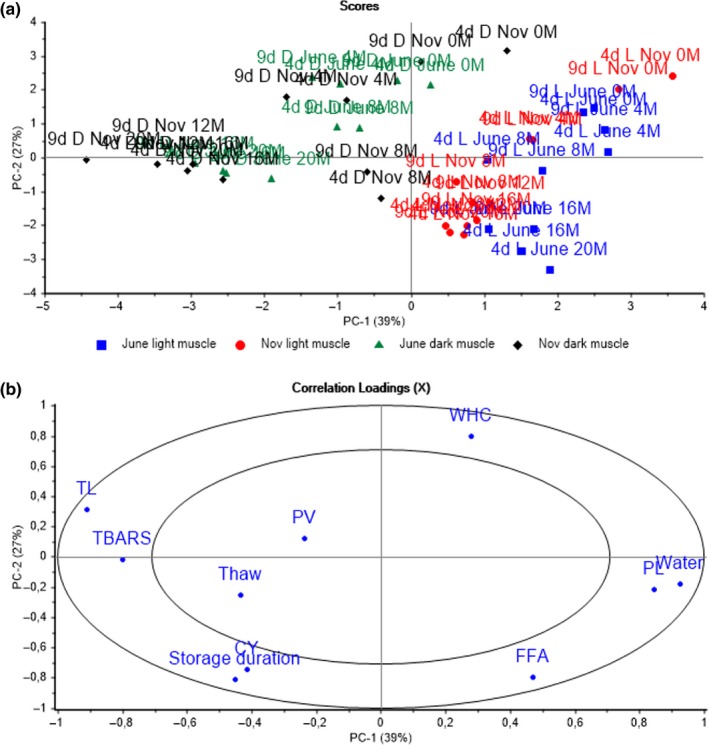
Scores (a) and correlation loadings (b) from the principal components analysis (PCA) of light and dark muscles of golden redfish fillets. The first number (4 and 9) and the second letter (d) indicate the processing delay in days. The letter L or D indicates the light and dark muscle. The number and the last letter (M) of the sample description indicate the frozen storage time in months. June and Nov indicate whether the fish were caught in June or November

A principal component analysis (PCA) was performed on the data to obtain an overview of the effects of the storage duration, raw material freshness, and season on the analyzed variables. The scores and correlation loadings from the first and second principal components (PCs) are shown in Figure 7. The PC1, representing 39% of the variance between the golden redfish samples, mostly indicated the differences in the chemical composition and lipid degradation of the light versus dark muscle types. The light muscle had a higher water content than the dark, while the dark muscle was characterized by a higher lipid content. PC2, representing 27% of the total variation, described the effects of storage duration on physical properties and lipid degradation. Thawing loss, CY, lipid hydrolysis, and oxidation increased with prolonged frozen storage, while the WHC decreased. As seen from the variable distribution on the loadings graph, the degradation of the light muscle was dominated by lipid hydrolysis, while the degradation of the dark muscle was dominated by lipid oxidation, in agreement with the mono‐variate analysis.

## 4 CONCLUSIONS

Season of capture affected both the nutritional value and stability of golden redfish. The light muscle of fish caught in November was richer in EPA and DHA than in the fish caught in June. The fish caught in November was also more unstable through frozen storage, due to a more unsaturated nature of the fatty acids present, indicating that special care needs to be applied during handling and treatment of golden redfish caught at this time. Fish processed and frozen 9 days postcatch had slightly higher thawing loss and yellowness values compared to fish processed on day 4, but no differences were found in other quality attributes.

The light muscle had a higher nutritional value than the dark muscle and is a good nutritional source for human consumption. However, the dark muscle was prone to lipid oxidation, which may have a negative influence on the more valuable light muscle. Removing the dark muscle by deep skinning could thus improve the quality and stability of redfish fillets. However, the dark muscle may be used for fish meal, fish oil production, or other valuable products for human consumption, leading to a more sustainable utilization of golden redfish.

## CONFLICT OF INTEREST

The authors declare that they do not have any conflict of interest.

## ETHICAL REVIEW

This study does not involve any human or animal testing.

## INFORMED CONSENT

Written informed consent was obtained from all study participants.
